# When seeing is learning: dynamic and interactive visualizations to teach statistical concepts

**DOI:** 10.3389/fpsyg.2015.00342

**Published:** 2015-03-25

**Authors:** David Moreau

**Affiliations:** Centre for Brain Research, University of AucklandAuckland, New Zealand

**Keywords:** visualization, mental imagery, spatial ability, didactics, monte carlo simulations, R, research methods, statistics

When Seeing is Learning: Dynamic and Interactive Visualizations to Teach Statistical Concepts “So, how would I use statistics in psychological research? First of all, descriptively.”

– Jacob Cohen (1990), p. 1310.

Jacob Cohen, one of the greatest statisticians of the twentieth century, reflected upon a problem many students of introductory statistics courses can relate to: the benefit of clear visual representations to understand statistical properties. Naturally, Cohen was not the first to touch upon this idea; in fact, he was echoing another exceptional statistician of the last century, John Tukey, who had emphasized the necessity of depicting data visually (Tukey, 1977). Decades later, this idea is still very contemporary—technologies have evolved, computing power has soared, but the perplexing nature of data analysis remains, especially for young scientists (Watts, 1991; Garfield and Ben-Zvi, 2007).

What advances in computing allow, however, is a fresh approach to circumvent the problem. Modern technologies offer an impressive panel of tools and support to teach statistics, in and outside the classroom. It is now possible for students to dynamically interact with their data, in order to understand the relative contribution of individual data points, variables, or parameters. For example, they can fairly easily introduce one data point at a time in an analysis and monitor how each observation refines the underlying model. Or observe how changes in one parameter result in differences in power calculations (Figure 1). Students can also access fairly abstract concepts such as randomness or sampling in a glimpse.

**Figure 1:**
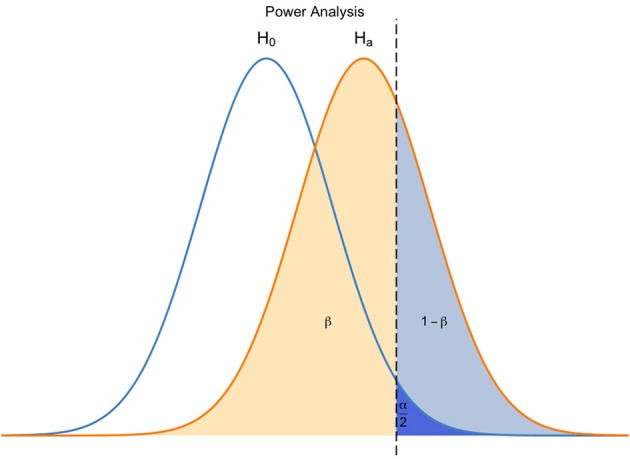
**Example of power analysis for a two-tailed symmetrical test**. With R packages such as shiny, students can modify any of the four quantities (α, β-1, ES, and N) and understand visually how their manipulation affects one another. Sliders can be added to the graph to facilitate manipulation (omitted here for clarity purposes).

Moreover, these advances come at a time when statistics is becoming a seductive and appealing field to students and the general public. In the New York Times and the Wall Street Journal, at Google and Facebook, the message is clear: statistics *is* sexy. Equally revealing, Hans Rosling, in a riveting and broadly-praised talk, advises not to be ashamed to say you are a statistician at a dinner party (Rosling, 2010). We have come a long way.

Yet how can we allow more students to grasp the critical concepts required to become statistically literate and apply data analysis methods adequately in current and future research programs? Many suggestions have been made, and this special research topic provides additional fascinating leads. In my opinion, one of the answers lies in the diversification of teaching contents and media. Decades of research have shown that individuals differ greatly in their ability to generalize, maintain and manipulate mental images. For example, while some need to constantly switch their focus of attention between the target figure and the possible answers in a mental rotation task, others can identify the correct answer in a blink, performing with remarkable accuracy in an effortless manner (Hegarty and Waller, 2005). It is therefore no coincidence one of the most eminent pioneers in the study of mental imagery has provided guidance on how to create effective visual presentations–he knows firsthand the tremendous variability among individuals when it comes to visualization, and the power of clear visual depictions to convey information accessible to everyone (Kosslyn, 2007). Interindividual variability in spatial ability also has important consequences in the way statistics should be taught in the classroom. An instructor cannot expect all students to extract the same information from a graphical depiction, or to be equally comfortable with complex representations of data. Because of these discrepancies, any effort to make visual content more accessible should be encouraged. Via dynamic and interactive graphics, today's technology allows students to visualize externally what they have difficulty representing mentally.

Dynamic and interactive visualizations also allow learning by active exploration—students can engage with their data, rather than try to understand them passively. Active engagement results in improved learning and verbal understanding (Bodemer et al., 2004) and is especially important since direct interaction with visual content facilitates the involvement of the motor system (Wraga et al., 2003), a component often present in highly-visual individuals and crucial to achieve deeper levels of understanding. Data exploration can then become a multi-sensory experience, setting the stage for profound and effective learning. Interactive visualizations are also more enjoyable, and in that respect possess prime value to motivate reluctant students (Papastergiou, 2009).

These advantages come at a cost. They require additional work beforehand on the teacher's part, to integrate components that are essential for valid, powerful pedagogical content. Graphics should include relevant content, but no more–too often, visual depictions contain more information than one can possibly process, resulting in cognitive overload and additional effort to consider information by chunks at a time (Lowe, 2003). For example, content from three-dimensional depictions could often be presented with separate two-dimensional plots, easier to comprehend. Just because a software offer the possibility of advanced graphics does not mean it is always the most appropriate choice. Simple, well-thought representations are often more effective. Directly related to this idea, changes in appearance should reflect new information, rather than diversification for esthetic purposes. When it comes to representing data, people expect changes in features to carry information (Kosslyn, 2007). Finally, visualizations need to be accessible to anyone, which means working toward user-friendly, non-discriminatory content. For example, graphics should avoid hues indiscernible by color-blind individuals. Wide accessibility also means opting for free software whenever possible, to guarantee sustained and undisrupted access. In this regard, graphical representations are made extremely easy by the growing popularity of statistical software such as R (R Core Team, 2014). Besides being free and open-source, allowing students to explore and play with their data from anywhere, R makes dynamic, interactive visualizations simple, with packages such as shiny, googleVis, and rCharts. R also lets creative instructors build *ad hoc* scripts and packages for their teaching purposes, thus enabling any type of data visualization.

Another definite asset of R and of the new generation of software pertains to the simplicity of simulating data. It is often crucial for students to make sense of a concept visually before transitioning to more sophisticated mathematical models. Despite the obvious advantages in understanding data, visual inputs can lack the general aspect of equations–they reflect a particular pattern of data at a specific time, yet to fully grasp most statistical concepts, students need to build an internal representation general enough to be useful in a wide range of situations. Simulations bridge that gap, as they help derive rules from the dynamic exploration of visual outputs. Other possibilities exist for implementing simulations, such as Java applets, but R has the remarkable advantage of including almost all a student needs in one single software.

In closing, it is a great time to be studying statistics—between online resources, MOOCs, free software and the appeal of data science to the academic and professional worlds, everything seems well-aligned to reward a curriculum emphasizing statistics. It is equally exhilarating to be teaching statistics—new technologies offer suitable tools to personalize content and reach more students. If any, the downside may be that there will soon be no excuse for imprecise knowledge or inaccurate applications of statistical techniques. As teachers and instructors embrace this technological revolution, a statistics-literate generation of psychologists will emerge, less prone to misuse statistics, and more likely to advance scientific knowledge while conveying clear, understandable messages to general audiences.

## Conflict of interest statement

The author declares that the research was conducted in the absence of any commercial or financial relationships that could be construed as a potential conflict of interest.

## Recommended online resources

https://developers.google.com/chart/interactive/docs/gallery

https://www.geogebra.org/

http://rcharts.io/

http://rpsychologist.com/

http://shinyapps.org/

http://shiny.rstudio.com/

http://showmeshiny.com/

http://swirlstats.com/
